# CHILDLESSNESS AND LONELINESS IN LATER LIFE? THE MODERATING ROLE OF REASONS FOR CHILDLESSNESS

**DOI:** 10.1093/geroni/igad104.2378

**Published:** 2023-12-21

## Abstract

Having children is a significant life event that causes major changes in social networks. Despite the fact that percentages of childlessness are on the rise worldwide, the connection between childlessness and loneliness in old age is still underresearched. Additionally, the scientific literature frequently draws a line between childlessness that is voluntary and involuntary, while a considerably gray area of reasons for childlessness is present between these two extremes of the continuum. The hypothesis of this study is that there is a moderating effect of reasons for childlessness on the link between childlessness (i.e., having children or not) and loneliness. A cross-sectional survey was created for this, containing questions on topics like social relationships, childlessness, and reasons for childlessness, as well as several validated measurement instruments, such as the De Jong Gierveld 11-item Loneliness Scale and the 3-item UCLA Loneliness Scale. A total of 500 older adults, at least 60 years old, participated in this study, with half of them being childless. The findings describe 1) the degree of social, emotional, and overall loneliness among older adults with and without children; 2) the reasons of childlessness among older adults; 3) the relationship between childlessness and current loneliness; and 4) whether the relationship between childlessness and loneliness is moderated by the reasons for childlessness. This study increases understanding of various aspects of loneliness among older persons in relation to childlessness, as well as the accompanying changes in the dynamics of social relations in later life.

